# Assessing the relationship between energy-related methane emissions and the burden of cardiovascular diseases: a cross-sectional study of 73 countries

**DOI:** 10.1038/s41598-023-40444-7

**Published:** 2023-08-19

**Authors:** Oliver Mendoza-Cano, Xóchitl Trujillo, Miguel Huerta, Mónica Ríos-Silva, Agustin Lugo-Radillo, Jaime Alberto Bricio-Barrios, José Clemente Rueda-Abad, Rebeca Yasmín Pérez-Rodríguez, Ana Luz Quintanilla-Montoya, Juan Manuel Uribe-Ramos, Valeria Argentina Mendoza-Olivo, Efrén Murillo-Zamora

**Affiliations:** 1 Faculty of Civil Engineering, University of Colima, km. 9 Colima-Coquimatlán Highway, 28400 Coquimatlán, Colima Mexico; 2 University Center for Biomedical Research, University of Colima, 25 de Julio Avenue 965, 28045 Colima, Colima Mexico; 3 CONAHCyT—University of Colima, University Center for Biomedical Research, 25 de Julio Avenue 965, 28045 Colima, Colima Mexico; 4 CONAHCyT — Faculty of Medicine and Surgery, Universidad Autónoma Benito Juárez de Oaxaca, Ex Hacienda de Aguilera S/N, Carr. a San Felipe del Agua, 68020 Oaxaca, Oaxaca Mexico; 5 Faculty of Medicine, University of Colima, Universidad Avenue 333, 28040 Colima, Colima Mexico; 6 Climate Change Research Program, National Autonomous University of Mexico, Scientific Research S/N, University City, 04510 Mexico City, Mexico; 7 Division of Natural and Exact Sciences, Department of Chemistry, University of Guanajuato, Noria Alta Unit, Col. Noria Alta S/N, 36050 Guanajuato, Guanajuato Mexico; 8 Unit of Clinical Epidemiology Research, Mexican Institute of Social Security, Lapislázuli Avenue 250, 28984 Villa de Álvarez, Colima Mexico

**Keywords:** Environmental sciences, Risk factors, Epidemiology

## Abstract

The energy industry significantly contributes to anthropogenic methane emissions, which add to global warming and have been linked to an increased risk of cardiovascular diseases (CVD). This study aims to evaluate the relationship between energy-related methane emissions and the burden of CVD, measured in disability-adjusted life years (DALYs), in 2019. We conducted a cross-sectional analysis of datasets from 73 countries across all continents. The analyzed datasets included information from 2019 on environmental energy-related methane emissions, burden of DALYs due to CVD. The age-standardized prevalence of obesity in adults and life expectancy at birth were retrieved. The relationship between the variables of interest was evaluated using multiple linear regression models. In the multiple model, we observed a positive linear association between methane emissions and the log-transformed count of DALYs related to CVD. Specifically, for each unit increase in energy-related methane emissions, the burden of CVD increased by 0.06% (95% CI 0.03–0.09%, *p* < 0.001). The study suggests that reducing methane emissions from the energy industry could improve public health for those at risk of CVD. Policymakers can use these findings to develop strategies to reduce methane emissions and protect public health.

## Introduction

Methane, a potent greenhouse gas, is emitted from various sources, including the energy industry through the production, processing, and transportation of fossil fuels^1–3^. Currently, energy-related industry emissions account for approximately 40% of total methane discharge. However, independent analysis conducted by the International Energy Agency (IEA) suggests that these emissions are 70% higher than what national governments have officially reported^4^.

Methane emission from these activities has been identified as a significant contributor to global warming and climate change^5^. However, the potential impact of methane emission on human health, particularly on cardiovascular health, has received limited attention^6^.

The link between environmental exposure to methane emissions and cardiovascular diseases (CVD) is a topic of growing interest in the scientific community^7^. The CVD, including coronary artery disease, stroke, and heart failure, are a leading cause of mortality worldwide^8^.

The burden of CVDs is striking, with an ever-increasing trend observed across diverse populations and age groups^9^. Contributing factors such as sedentary lifestyles, unhealthy diets, tobacco use, obesity, and hypertension have further fueled the epidemic^10–12^.

Environmental exposures to air pollution, including particulate matter and nitrogen oxides, have been associated with an increased risk of CVD^13,14^. Several potential mechanisms may link exposure to methane emissions and CVD. For example, methane emission may contribute to the formation of ozone and fine particulate matter, both of which have been linked to cardiovascular risk^15^. Methane may also contribute to the production of reactive oxygen species, leading to oxidative stress and inflammation^16^, both of which are implicated in the development of these group of illnesses^17^.

Due to the substantial contribution of the energy industry to methane emissions and the potential impact on cardiovascular health, a comprehensive understanding of this relationship is crucial. This understanding can inform policies aimed at reducing methane emissions and safeguarding public health. Thus, the objective of this study was to investigate the potential association between energy-related methane emissions and the burden of CVD using an ecological approach during 2019.

## Methods

We conducted a cross-sectional analysis using an ecological approach, utilizing three publicly available datasets. Firstly, we obtained data on energy-related methane emissions, measured in tons per year (TPY), for the year 2019 from the Global Methane Tracker provided by the IEA^18^.

Secondly, we obtained information on the total burden related to CVD, measured in disability-adjusted life years (DALYs) and for the year 2019, from the Global Burden of Diseases, Injuries, and Risk Factors Study (GBD) 2019 update^19^. The codes (I00–I99, International Classification of Diseases, Tenth Revision[ICD-10]), also for the year 2019, were included.

Finally, we obtained data on the age-standardized prevalence of obesity at the country level, defined as a body mass index 30 or above in adults, from the Global Health Observatory (GHO)^20^. The data retrieved from the aforementioned repository represented the most recent available estimates, specifically for the year 2017. The life expectancy at birth (in years) for the year 2019 was sourced from the World Bank^21^. These two variables (age-standardized prevalence of obesity and life expectancy at birth) were considered as potential confounding factors. We excluded nations that lacked any of the estimators of interest.

We calculated summary statistics, which included the Spearman rank-order correlation coefficient (rho) and 95% confidence intervals (CI). The *p* value from the Shapiro-Francia test was < 0.001 for the distribution of methane emissions and DALY count in the analyzed countries.

Additionally, we used linear regression models to assess the relationship between the variables of interest, creating a total of four models. The first model examined the correlation between DALY count and methane emissions, the second and third models included the age-standardized prevalence of obesity and life expectancy (respectively and as confounding variable), and the fourth model was a multiple linear regression model that encompassed methane emissions, obesity prevalence and life expectancy at birth as exposure variables.

We computed the studentized residuals from the multiple model and identified heteroscedasticity (W′ = 0.79, V′ = 15.53, *p* < 0.001). Therefore, we proceeded to perform a log transformation of the dependent variable and ran the multiple regression model using this transformed variable. The distribution of the studentized residuals from this new model was found to be homogeneous (W′ = 0.99, V′ = 0.73, *p* = 0.730). The results presented below are derived from the log-transformed model.

Since we exclusively analyzed publicly available data for academic purposes, we did not obtain approval from an ethics committee. However, we ensured that our study adhered to ethical principles and guidelines.

### Ethics declarations

As we exclusively analyzed publicly available data for academic purposes, we were not required to obtain approval from an ethics committee.

## Results

The African countries included in the study were Angola, Benin, Botswana, Eritrea, Equatorial Guinea, Ghana, Guinea, Kenya, Libya, Namibia, Niger, Nigeria, Senegal, Somalia, South Africa, Sudan, Tunisia, and Togo. The Asian countries included were Azerbaijan, Bahrain, Bangladesh, Egypt, Indonesia, Iraq, Israel, Jordan, Kazakhstan, Kuwait, Lebanon, Malaysia, Oman, Pakistan, Philippines, Qatar, Saudi Arabia, Thailand, Turkmenistan, United Arab Emirates, Uzbekistan, and Yemen.

Europe was represented by Denmark, Estonia, France, Germany, Italy, Netherlands, Norway, Poland, Romania, Slovenia, Sweden, and the United Kingdom). A total of 10 American nations were included (namely Argentina, Brazil, Canada, Cuba, Ecuador, Guyana, Mexico, Paraguay, Trinidad and Tobago, and Uruguay) and Oceania was the least represented (Australia and New Zealand).

**Figure 1:**
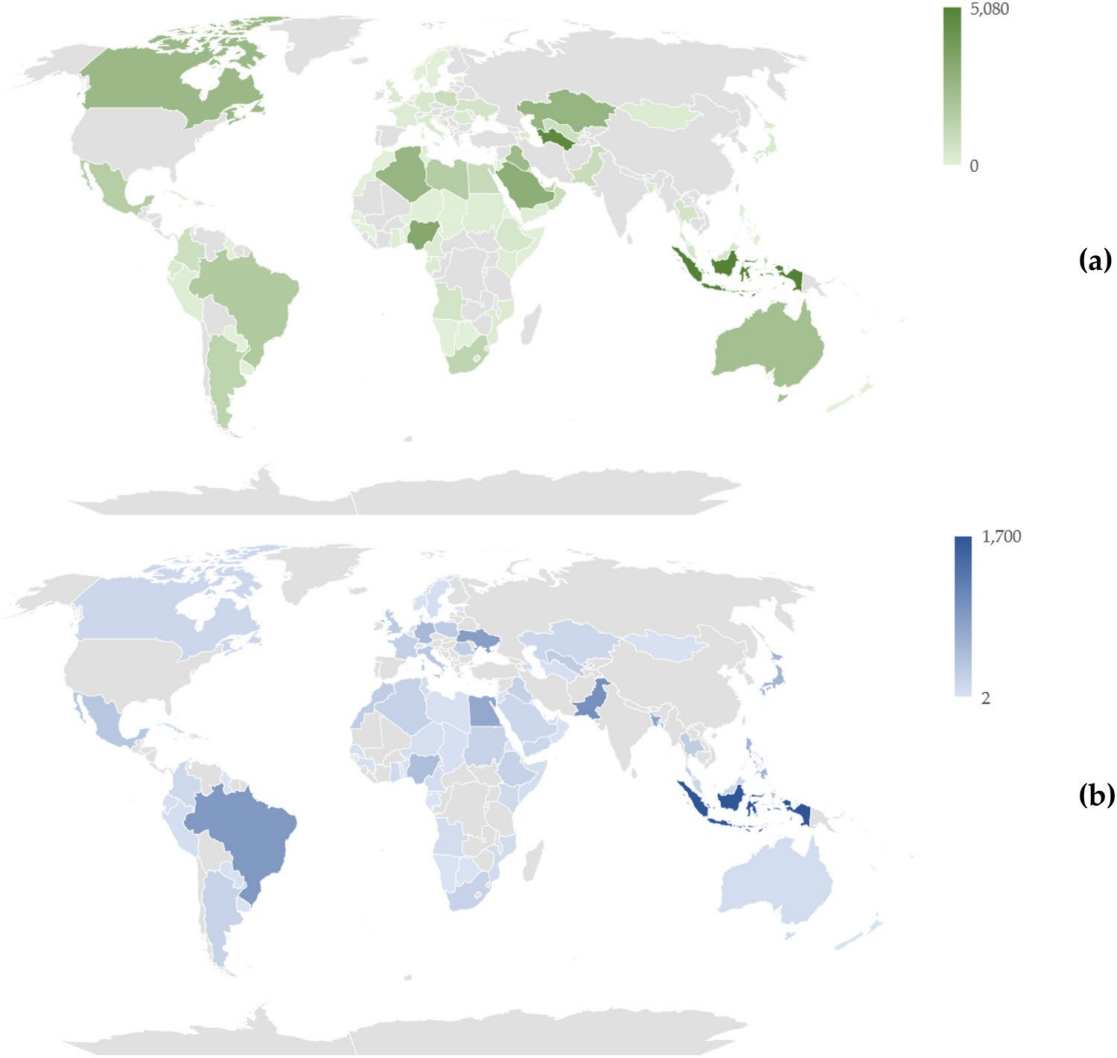
Energy-related methane emissions (tons per year) and total disability-adjusted life years (× 10^4^) attributable to cardiovascular diseases in 73 countries, 2019. *Note*: The choropleth maps presented above were created by the authors using the specialized tool provided by Microsoft Excel 365 (Microsoft Corporation; Redmon, WA, USA).

In terms of energy-related methane emissions, we found that the median value was 222.1 TPY (interquartile range: 41.0 to 894.8 TPY). However, there was a wide range of emissions, with some countries reporting as low as 0.01 TPY (e.g. Guinea) and as high as 5,080.4 TPY (e.g. Indonesia). Figure 1 displays the methane emissions (1a) and total DALY (× 10^4^) attributable to CVD (1b) in the analyzed countries in 2019.

Regarding the burden of CVD, we observed significant variation across nations, with total DALYs ranging from 24,771 in Equatorial Guinea to 16,511,184 in Indonesia. The median count of DALYs per country was 718,967.

In our analysis of obesity prevalence, we found a median age-standardized prevalence of 20.3% (interquartile range: 9.3–27.2%). Interestingly, Asian countries showed higher rates of obesity compared to other regions, with Kuwait reporting the highest prevalence (37.9%) and Bangladesh reporting the lowest (3.6%). The mean life expectancy at birth in the analyzed countries was 73.0 ± 7.7 years, ranging from 52.9 years (Nigeria) to 84.4 years (Japan).

We identified a significant positive correlation between methane emissions and DALYs attributed to CVD (Spearman’s rho = 0.53, 95% CI 0.34 to 0.68; *p* < 0.001), indicating that higher methane emissions are associated with a greater burden of CVD. However, we did not observe a significant correlation between total DALYs and the age-standardized prevalence of obesity in adults (Spearman’s rho =  − 0.16, 95% CI − 0.37 to 0.08; *p* = 0.185), nor with life expectancy at birth (Spearman’s rho =  − 0.03, 95% CI − 0.26 to 0.20; *p* = 0.780).

In our multiple regression analysis (Table 1), we identified a significant association between methane emissions and the total log-transformed DALYs related to CVD. Specifically, for each unit increase in energy-related methane emissions (measured as a continuous variable), the CVD burden increased by 0.06% (95% CI 0.03–0.09%). Additionally, the log-transformed burden of CVD showed a negative association with the prevalence of obesity in our multiple model ($e^{\beta }$ = 0.93, 95% CI 0.89–0.97, *p* = 0.002). As depicted in Table 1, each additional year of life expectancy at birth corresponded to a 6% increase in the CVD burden (95% CI 1–11%, *p* = 0.044). The coefficient of determination (R^2^) and adjusted R^2^ were 0.242 and 0.209, respectively.

**Table 1:** Relationship between analyzed variables and log-transformed CVD-related burden in 2019.

Variable	$e^{\beta }$(95% CI), *p*	
	Bivariate analysis	Multiple analysis
Energy-related methane emissions (TPY)	1.0005 (1.0001–1.0008), 0.002	1.0006 (1.0003–1.0009), < 0.001
Age-standardized prevalence of obesity in adults	0.97 (0.94–1.01), 0.141	0.93 (0.89–0.97), 0.002
Life expectancy at birth (years)	0.99 (0.95–1.05), 0.962	1.06 (1.01–1.11), 0.044

(1) The burden of CVD was evaluated using the total count of disability-adjusted life years (DALY); (2) The coefficients in the multiple model were adjusted for the variables listed in the table.

*CVD* Cardiovascular diseases, *CI* Confidence interval, *TPY* Tons per year.

## Discussion

In this study, we conducted an ecological analysis of data from 73 countries to examine the relationship between energy-related methane emissions and the burden of CVD in 2019. Our results indicate that methane emissions are significantly associated with the burden of CVD in the analyzed nations. Specifically, we found a positive correlation between methane emissions and the total DALYs attributed to CVD, indicating that higher methane emissions are associated with a greater burden of CVD. These results are in line with previous research linking air pollution and cardiovascular disease^22–25^.

It is worth noting the methodological limitations of ecological studies when interpreting the results. Our analysis is based on country-level data, which may not accurately reflect the individual-level exposure and health outcomes. Other factors not accounted for in our study, such as lifestyle and genetic factors, could also contribute to the development of CVD^26^.

In the energy industry, methane emissions are primarily generated through the extraction, production, and distribution of natural gas, which is composed mainly of methane. Methane is released into the atmosphere during the drilling, completion, and production of natural gas wells, as well as during the processing, storage, and transportation of natural gas^27^.

During the drilling process, methane can escape through leaks in the well casing or from venting and flaring of natural gas^28^. Methane can also be released during hydraulic fracturing, or fracking, when high-pressure fluids are injected into shale rock to release trapped natural gas^29^. Methane emissions can also occur during the processing and transportation of natural gas through pipelines, compressors, and other equipment^30^.

Additionally, methane can be released during coal mining operations when methane is trapped in coal seams and is released as the coal is mined^31^. Methane emissions can also occur during oil production when methane is co-produced with oil and is released through venting or flaring^32^.

The IEA, from where our data were obtained, reports a wide range of energy-related methane emissions registered across countries. This high variability could be attributed to differences in energy production and consumption patterns, as well as differences in the regulation and implementation of emission reduction policies^33^. For instance, Indonesia reported the highest methane emissions, which could be attributed to its large coal and oil industries, while Guinea reported the lowest emissions, which could be due to its low levels of industrialization.

Methane can enter the body through inhalation or ingestion^34^. Inhalation of methane can occur through breathing contaminated air, such as in occupational settings where workers are exposed to high levels of methane, or in areas with high levels of methane emissions, such as landfills or natural gas extraction sites^35^. Once inhaled, methane can be absorbed into the bloodstream through the lungs, where it can then be transported to other organs in the body.

Ingestion of methane can occur through the consumption of contaminated food or water^36,37^. Methane can be produced in the digestive system of certain animals, such as cows and sheep, and can be released into the environment through their excrement^38^. Contaminated food or water can contain high levels of methane, which can then be ingested and absorbed into the bloodstream through the digestive system.

Several mechanisms may be involved in the pathogenesis of CVD that are influenced by exposure to methane gas. Methane exposure has been shown to increase oxidative stress^39^, inflammation, and vascular dysfunction^40^, all of which are key risk factors for CVD.

Within the cells, methane can interact with mitochondria and may disrupt their function and compromise the electron transport chain, leading to the leakage of electrons and the generation of reactive oxygen species (ROS)^16^. The accumulation of ROS can overwhelm the endogenous antioxidant defense system, comprising enzymes such as superoxide dismutase, catalase, and glutathione peroxidase^41^. As a result, the balance between ROS production and antioxidant capacity is disrupted.

Oxidative stress and inflammation are closely interconnected processes that play critical roles in human health and disease^42^. Inflammation seems to be a key risk factor for cardiovascular diseases, as chronic inflammation can lead to the development of atherosclerosis^43,44^. Methane exposure has been shown to increase the production of pro-inflammatory cytokines and other markers of inflammation in the cardiovascular system, which can contribute to the development of atherosclerosis and other cardiovascular diseases^45^.

To the best of our knowledge, there are no published data estimating the attributable risk of methane emissions on CVD-related burden of disease. However, a recent and interesting publication considered that 2 out of 10 deaths due to non-communicable diseases may be attributed to ambient air pollution^46^. The CVDs that may arise, at least in part, from exposure to environmental methane emissions and other air pollutants include coronary heart disease, stroke, and heart failure^7^.

In our study, we observed significant variation in the burden of CVD across nations, with some countries reporting much higher DALY counts compared to others. This variation could be attributed to differences in CVD risk factors, including hypertension^47^, smoking^48,49^, and physical inactivity^50^, as well as differences in healthcare infrastructure and access to healthcare services^51,52^. This highlights the importance of understanding the unique socio-economic and environmental factors that may contribute to CVD burden in different regions.

We used age-standardized prevalence of obesity as an adjustment variable, given its established link to CVD^53^. However, we observed a significant but inverse relationship in the 73 countries analyzed.

Life expectancy at birth (measured in years) was included as a potential confounding variable in our study. It was chosen as a proxy for a range of unobserved characteristics present within a population. Life expectancy at birth is known to be influenced by various factors, such as healthcare access, socioeconomic conditions, environmental quality, and lifestyle behaviors, among others^54,55^. These factors may have indeterminate effects on the outcome of interest in our research.

However, we acknowledge that including obesity prevalence and life expectancy does not eliminate all potential confounding factors in a study of our nature. Therefore, we emphasize that the limitations associated with an ecological approach must be carefully considered when interpreting the findings presented.

## Conclusions

Our study provides valuable insights into the relationships between energy-related methane emissions and the burden of CVD. These findings could inform the development of public health policies aimed at reducing the burden of CVD and promoting sustainable development. Further studies are needed to explore the mechanisms underlying these relationships and to identify effective interventions in diverse populations.

## Data Availability

The analyzed data are available in four public repositories (all accessed on June 26, 2023): energy-related methane emissions in 2019 from the Global Methane Tracker by the International Energy Agency (IEA), which can be found at https://api.iea.org/methane/comparison?source=IEA&csv=true; the total burden related to CVD from the Global Burden of Diseases, Injuries, and Risk Factors Study (GBD) in 2019, available at https://vizhub.healthdata.org/gbd-results/; the country-level age-standardized prevalence of obesity in adults in 2017 from the Global Health Observatory (GHO), available at https://www.who.int/data/gho/data/indicators/indicator-details/GHO/prevalence-of-obesity-among-adults-bmi-=-30-(age-standardized-estimate)-(-); and the life expectancy at birth for the analyzed countries in 2019, and which is available at https://data.worldbank.org/indicator/SP.DYN.LE00.IN.

## Abbreviations

CVD: Cardiovascular diseases
DALY: Disability-adjusted life years
CI: Confidence interval
TPY: Tons per year
IEA: International Energy Agency
GBD: Global Burden of Diseases, Injuries, and Risk Factors Study
ICD-10: International Classification of Diseases, Tenth Revision
GHO: Global Health Observatory
